# Effect of the expression and knockdown of citrate synthase gene on carbon flux during triacylglycerol biosynthesis by green algae *Chlamydomonas reinhardtii*

**DOI:** 10.1186/1471-2091-14-38

**Published:** 2013-12-30

**Authors:** Xiaodong Deng, Jiajia Cai, Xiaowen Fei

**Affiliations:** 1 School of Science, Hainan Medical College, Haikou 571101, China; 2 Key Laboratory of Tropical Crop Biotechnology, Ministry of Agriculture, Institute of Tropical Bioscience and Biotechnology, Chinese Academy of Tropical Agricultural Science, Haikou 571101, China

**Keywords:** Citrate synthase, Triacylglycerol biosynthesis, RNAi interference, Overexpression, *Chlamydomonas reinhardtii*, Nitrogen deprivation

## Abstract

**Background:**

The regulation of lipid biosynthesis is essential in photosynthetic eukaryotic cells. This regulation occurs during the direct synthesis of fatty acids and triacylglycerols (TAGs), as well as during other controlling processes in the main carbon metabolic pathway.

**Results:**

In this study, the mRNA levels of *Chlamydomonas* citrate synthase (CrCIS) were found to decrease under nitrogen-limited conditions, which suggests suppressed gene expression. Gene silencing by RNA interference (RNAi) was conducted to determine whether *CrCIS* suppression affected the carbon flux in TAG biosynthesis. Results showed that the TAG level increased by 169.5%, whereas the CrCIS activities in the corresponding transgenic algae decreased by 16.7% to 37.7%. Moreover, the decrease in CrCIS expression led to the increased expression of TAG biosynthesis-related genes, such as acyl-CoA:diacylglycerol acyltransferase and phosphatidate phosphatase. Conversely, overexpression of *CrCIS* gene decreased the TAG level by 45% but increased CrCIS activity by 209% to 266% in transgenic algae.

**Conclusions:**

The regulation of *CrCIS* gene can indirectly control the lipid content of algal cells. Our findings propose that increasing oil by suppressing *CrCIS* expression in microalgae is feasible.

## Background

Considering that fossil fuel resources are limited, the importance of conserving energy and saving the environment is gaining increased interest. Microalgae biodiesel, a crucial renewable biomass energy that uses solar energy to fix CO_2_ into biomass, is the most promising alternative to fossil fuels. However, studies on lipid metabolism in eukaryotic, single-celled, photosynthetic microalgae are limited compared with those on oil crops. Basic knowledge on microalgae is less than that of crops, such as rice, wheat, and corn. With the intensification of microalgae-derived biodiesel research at the global scale, more researchers are focusing on the mechanism underlying the formation of high lipid production and high cell-density cultures. These processes are crucial in genetic strain improvement, as well as in the future cultivation of commercial and industrial microalgae.

The substrate competition hypothesis was proposed by Sugimoto et al. and Chen et al. [1-3]. According to this hypothesis, the lipids and proteins of seeds compete for the same substrate, i.e., pyruvate, which is a product of glycolysis. Phosphoenolpyruvate carboxylase (PEPC; EC4.1.1.31) catalyzes the formation of oxaloacetate (OAA) from phosphoenolpyruvate (PEP), and then enters the tricarboxylic (TCA) cycle to provide the substrate and energy needed for protein synthesis. Meanwhile, acetyl–CoA carboxylase (ACCase) catalyzes the formation of acetyl coenzyme A from pyruvate, and then enters the fatty acid synthesis pathway. Hence, photosynthetic carbon flux tends to synthesize proteins or lipids depending on the relative activity of PEPC and ACCase. Using antisense RNA technology to suppress PEPC activity, they obtained stable, high-oil rape seed lines and high-oil soybean lines whose lipid contents increased by 6.4% to 18%. Citrate synthase (CIS; E.C. 2.3.3.1) is common within various organisms. CIS isozymes exhibit diverse subcellular localization patterns, and are involved in crucial physiological metabolic pathways. CIS, which is located in the mitochondria, is a rate-limiting enzyme during the first step of the TCA cycle. Through this step, CIS forms citroyl–CoA by catalyzing the reaction of acetyl–CoA. Currently, biological, functional, and applied research regarding CIS in plants mainly focus on the following aspects. First, crops are stimulated to secrete citric acids through CIS gene overexpression, which can activate hard-soluble P in soil to increase soil phosphorus utilization. In this context, Tong et al. cloned a CIS gene from rape and detected the gene expression under stressful conditions [4]. Correspondingly, Hu et al. bred a low-phosphorus resistant stain by overexpressing CIS gene into rice; the rice exhibited rapid root growth and high secretion of citric acid [5]. Second, CIS may increase cell resistance to aluminum toxicity. Citric acid forms a complex with Al^3+^. This complex exhibits either low toxicity or non-toxicity. The overexpression of CIS in alfalfa evidently strengthens the tolerance of the plant to acidic soil and aluminum toxicity [6]. Third, CIS expression is related to organic acid accumulation during fruit ripening [7]. Under this aspect, Zhang et al. provided evidence that the citric acid content of pineapple fruit is positively correlated with CIS activity during pineapple maturation [8]. Studies on CIS in microalgae are rarely reported compared with those in vascular plants and animals. As early as the 1970s, Barrie et al. published a study on CIS in microalgae. They detected CIS activity in four kinds of Cyanophyta, whose activity is inhibited by factors such as α-ketoglutaric acid, nicotinamide adenine dinucleotide phosphate, and ATP; this inhibition is speculated to lead fixed carbon flux to lipid synthesis [9].

We have confirmed that existing studies have not demonstrated any evidence that *CIS* gene expression and regulation are related to cellular lipid accumulation. Accordingly, this study aimed to determine whether such relationship exists. The mRNA abundances of *CrCIS* and lipid accumulation were detected under plus/minus nitrogen conditions in *Chlamydomonas reinhardtii* CC124. Knockdown by RNAi and overexpression of *CrCIS* gene were then performed in *Chlamydomonas* to determine the effect of overexpression or inhibition of *CrCIS* on cellular carbon flux and lipid accumulation. Furthermore, the results of this study can contribute in establishing the relationship of lipid accumulation with carbon flux distribution.

## Results

### Cloning of *CrCIS* gene and bioinformatics analysis

**Figure 1:**
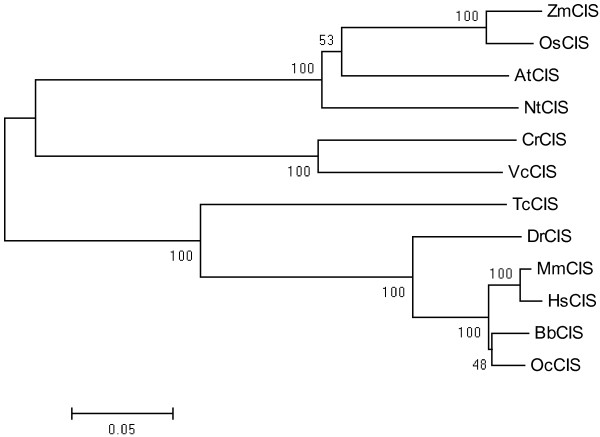
**Clustering analysis of citrate synthase orthologous genes in *C. reinhardtii *and other species.** AtCIS: *Arabidopsis thaliana* citrate synthase(AAM62868); BbCIS: Bubalus bubalis citrate synthase (AEO51018); DrCIS: Danio rerio citrate synthase (NP_955892); TcCIS :*Tribolium castaneum* citrate synthase (XP_970124); HsCIS:Homo sapiens citrate synthase (BAG58964); MmCIS: Mus musculus citrate synthase (NP_080720); NtCIS: Nicotiana tabacum citrate synthase (CAA59008); OcCIS: *Oryctolagus cuniculus* citrate synthase (XP_002711121); OsCIS: *Oryza sativa* citrate synthase (NP_001068031); VcCIS:Volvox carteri citrate synthase (XP_002948056.1); ZmCIS: Zea mays citrate synthase (NP_001132846).

An approximately 1500 bp DNA fragment of *CrCIS* gene full-length CDS was amplified, cloned, and sequenced, exhibiting 100% homology with Chlamydomonas *CIS* gene (Protein ID194915). Using the BLAST programs and the Chlamydomonas *CIS* gene as entries, we obtained the *CrCIS* orthologous genes from the NCBI database. The amino acid sequence alignment of the *CIS* orthologous genes was created using ClustalW (http://www.genome.jp/tools/clustalw/). The phylogenetic tree of the *CIS* orthologous genes is presented in Figure 1. All listed *CIS* orthologous genes contained the citrate synthase function domain. The predicted subcellular location of CrCIS (by Euk-mPLoc 2.0) was within the mitochondrion (http://www.csbio.sjtu.edu.cn/bioinf/euk-multi-2/).

### mRNA level of *CrCIS* under N sufficient and N limited conditions

**Figure 2:**
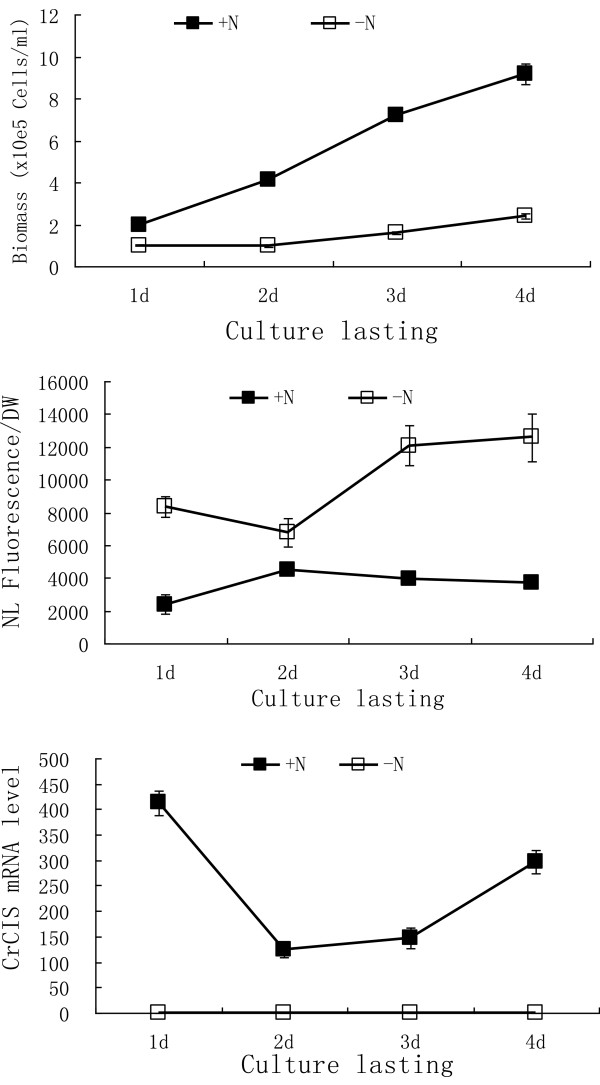
**The mRNA abundance of *CrCIS *gene and corresponding biomass and lipid content of the cells in HSM and HSM-N media.** The mRNA levels were analyzed through RT-PCR of *C. reinhardtii CC425* samples grown in the indicated media for 1, 2, 3, or 4 d. +N: cells cultivated in N sufficient HSM medium; -N: cells cultivated in N free HSM medium.

To determine the mRNA levels of the *CrCIS* under N-sufficient and N-limited conditions, 50 mL of cultivated Chlamydomonas (2 × 10^6^ cells/mL) was collected through centrifugation. After washing with HSM-N medium, the cells were suspended and half of them were transferred to a new 50 mL of HSM and HSM-N medium for further cultivation. Algal cells harvested at 24, 48, 72, or 96 h were used for RNA extraction. We quantitatively determined the expression of *CrCIS* gene in these samples through reverse transcription followed by real-time polymerase chain reaction (PCR). Results presented in Figure 2 exhibit the difference in lipid accumulation of cells in the two conditions; cells accumulated three to six times more lipids under the N-limited condition than under the N-sufficient condition. Interestingly, *CrCIS* mRNA was undetectable under the N-limited condition. Thus, we further determined whether the decline in the mRNA of *CrCIS* levels influenced the increase in lipid accumulation.

### Silencing of *CrCIS* gene increases triacylglycerol (TAG) content in *C. reinhardtii*

**Figure 3:**
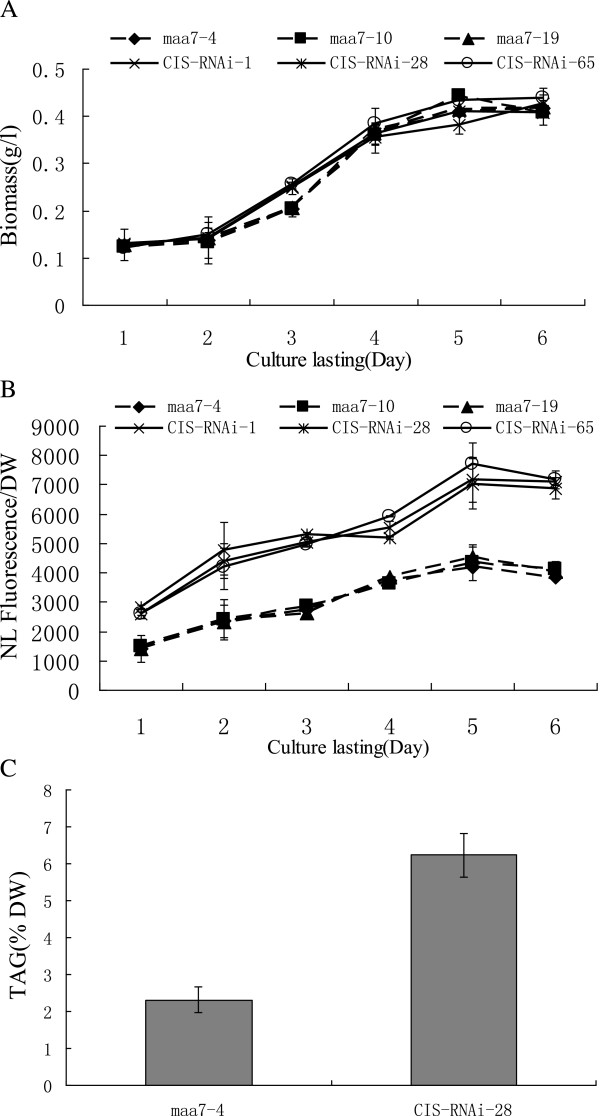
**The biomass and lipid content detected through Nile Red staining method and TAG level in *CrCIS *RNAi transgenic *C. reinhardtii*. A)** The growth curve of *CrCIS* RNAi transgenic algae. **B)** The lipid content detected Nile Red staining method of *CrCIS* RNAi transgenic algae. **C)** TAG level detected by GC/MS after the strains cultivated for six days. Maa7-4(10,19): pMaa7IR/XIR transgenic algae strains; CIS-RNAi-1(28,65): pMaa7IR/CrCISIR transgenic algae strains.

**Figure 4:**
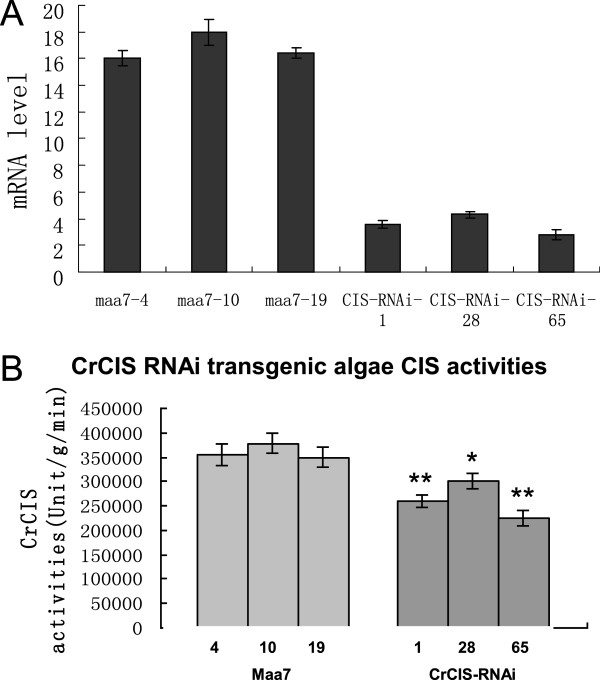
**Comparison of the mRNA abundance and enzyme activity of *CrCIS *in transgenic *C. reinhardtii*. (A)** The mRNA abundance of *Cr*CIS in transgenic *C. reinhardtii*; **(B)** CIS activity in *CrCIS* RNAi transgenic *C. reinhardtii*.Maa7-4(10,19), pMaa7IR/XIR transgenic algae strains; CIS-RNAi-1(28,65), pMaa7IR/CrCISIR transgenic algae strains. Statistical analysis was performed using SPSS statistical software. Significance is indicated as *P < 0.05, **P < 0.01.

To determine the relationship between *CrCIS* expression and lipid accumulation, we examined the effects of the artificial silencing of *CrCIS* gene on the lipid content of *C. reinhardtii*. Based on the *CrCIS* (194915) sequences of the gene retrieved from the JGI *C. reinhardtii* v4.0 database, we designed primers (Additional file 1: Table S1) to amplify the fragment of the coding region of *CrCIS*. The DNA fragments were subcloned and then used to generate *CrCIS* RNAi constructs pMaa7IR/CrCIS IR. More than 120 positive transformants were obtained after transforming the silencing construct into *C. reinhardtii* CC425. Three transgenic algae were selected to measure the lipid content and mRNA levels of the targeted gene. Strains transformed with the vector pMaa7IR/XIR were used as controls. In cells harboring the *CrCIS* construct, analysis of the transgenic lines through the Nile red fluorescence method indicated the increase of the lipid content by 75.0% to 92.6% (Figure 3B) after six days of cultivation. The TAG level of the transgenic strain CIS-RNAi-28 increased by 169.5% compared with the control (Figure 3C). To evaluate the effectiveness of the RNAi construct, the abundance of target gene-specific mRNA through real-time PCR in transgenic algae was analyzed. The *CrCIS* mRNA abundance decreased by 72.8% to 81.0% (Figure 4A), indicating high-efficiency silencing by these constructs.

**Figure 5:**
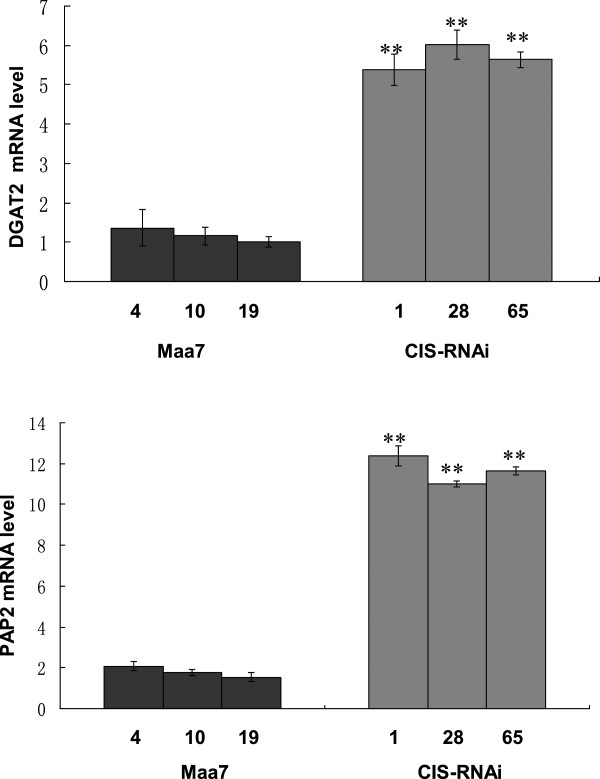
**The mRNA abundance of *DGAT2 *and *PAP2 *gene in *CrCIS *RNAi transgenic algae strains.** Maa7-4(10,19): pMaa7IR/XIR transgenic algae strains; CIS-RNAi-1(28,65): pMaa7IR/CrCISIR transgenic algae strains. Statistical analysis was performed using SPSS statistical software. Significance is indicated as *P < 0.05, **P < 0.01.

The enzyme activities of CrCIS of transgenic strains were tested. Compared with that of the wild strains, the CrCIS activity of *CrCIS* RNAi transgenic strains decreased by 16.7% to 37.7% (Figure 4B). Subsequently, the mRNA levels of *DGAT2* (acyl-CoA:diacylglycerol acyltransferase) (Deng et al. [10]) and *PAP2* (phosphatidate phosphatase) genes are directly related to lipid synthesis, and were measured in *CrCIS* RNAi transgenic strains. This result showed that the mRNA levels of *DGAT2* and *PAP2* genes increased in both transgenic strains (Figure 5). These outcomes indicated the decrease in enzyme activities caused by the silencing of *CrCIS* gene, thereby slowing down cellular TCA. The silencing of the *CrCIS* gene indirectly partitioned photosynthetic carbon into fatty acid and lipid synthesis by increasing *DGAT2* and *PAP2* gene expression.

**Figure 6:**
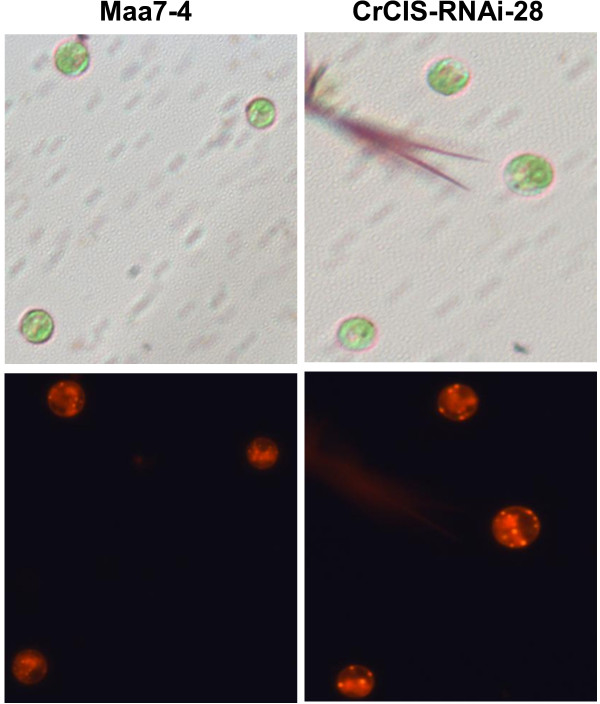
**Microscopic observations of *CrCIS *RNAi transgenic *C. reinhardtii*.** After 4 days of cultivation in HSM medium, more oil droplets of *CrCIS* RNAi transgenic algae had been found. Maa7-4, pMaa7IR/XIR transgenic algae strain number 4; CIS-RNAi-28, pMaa7IR/CrCISIR transgenic algae strain number 28.

Similar results were obtained in Nile Red staining. More oil droplets were found in *CrCIS* RNAi transgenic algae compared with those in pMaa7IR/XIR transgenic algae as determined by microscopic analysis (Figure 6) and counting the number of oil droplets of transgenic strains (Additional file 1: Table S3). These data indicated the increase in cell lipid content through the regulation of *CrCIS* gene expression despite different metabolic pathways.

### Overexpression of *CrCIS* reduced the lipid content of *C. reinhardtii*

**Figure 7:**
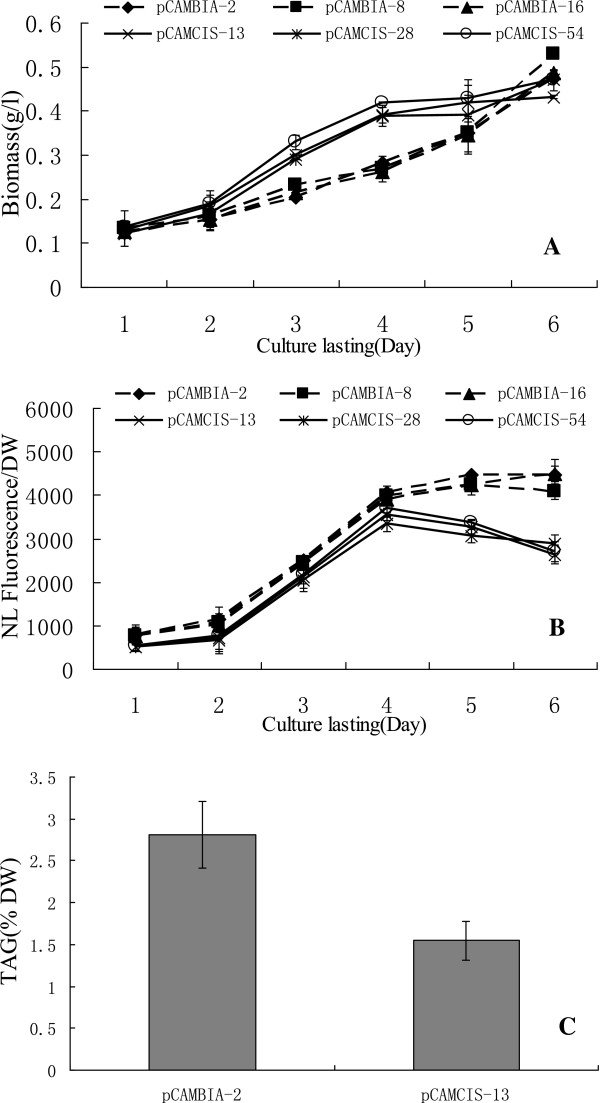
**The biomass and lipid content detected through Nile Red staining method and TAG level of over-expression of *CrCIS in *transgenic algae in HSM medium. A)** The growth curve of *CrCIS* transgenic algae. **B)** The lipid content detected through Nile Red staining method of *CrCIS* transgenic algae. **C)** TAG level detected through GC/MS after the strains cultivated for 6 days. pCAMBIA-2(8,16): pMCAMBIA1302 transgenic algae strains; pCAMCIS-13(28,54): pCAMCIS transgenic algae strains.

**Figure 8:**
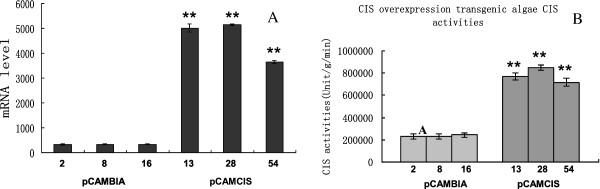
**Comparison of the mRNA abundance and enzyme activity of *CrCIS *in transgenic *C. reinhardtii*. A)** The mRNA abundance of *CrCIS* transgenic algae; **B)** The CIS enzyme activity of CrCIS transgenic algae. pCAMBIA-2(8,16): pMCAMBIA1302 transgenic algae strains; pCAMCIS-13(28,54): pCAMCIS transgenic algae strains. Statistical analysis was performed using SPSS statistical software. Significance is indicated as *P < 0.05, **P < 0.01.

**Figure 9:**
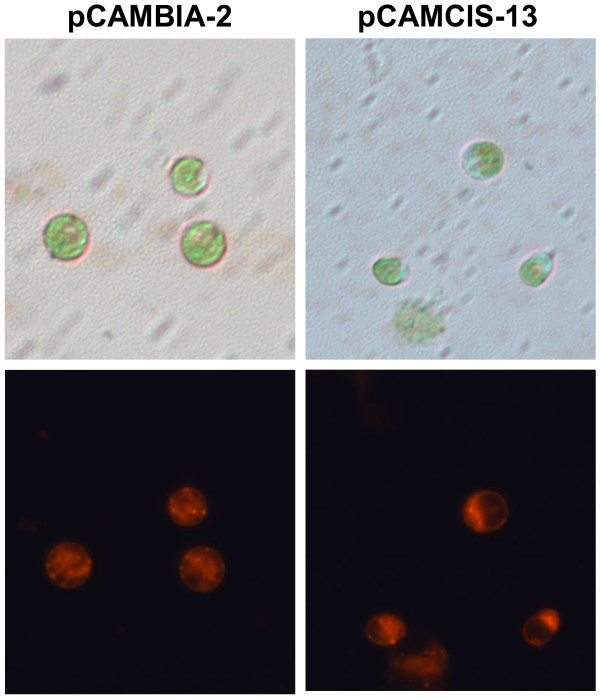
**Lipid content in transgenic algae line detected through Nile red staining method.** After 4 days cultivation in HSM medium, less oil droplets of *CrCIS* transgenic algae had been found. pCAMBIA-2: pMCAMBIA1302 transgenic algae strain number 2; pCAMCIS-13: pCAMCIS transgenic algae strain number 13.

The increase in lipid content caused by RNAi silencing of *CrCIS* suggested the effect of the expression of these genes on the biosynthesis of triglycerides in *C. reinhardtii*. Thus, we determined whether *CrCIS* overexpression can reduce the lipid content of *C. reinhardtii*. Vector pCAMCIS, which expressed *CrCIS* gene from the CAMV 35S promoter, was introduced into *C. reinhardtii*. The lipid contents and growth rate of three randomly selected transgenic algae were detected in each transgenic algae line. Overexpression of *CrCIS* gene increased growth rate in the early stages from day two to day five. Moreover, overexpression of *CrCIS* decreased the lipid content compared with that of the control pCAMBIA1302 transgenic algae lines. For example, after six days of growth in HSM medium, the lipid contents of *CrCIS* overexpressing transgenic lines determined by the Nile Red fluorescence method decreased by 33.8% to 39.5% (Figure 7B). The TAG level of the transgenic strain pCAMCIS-13 decreased by 45% compared with the control (Figure 7C). Compared with pCAMBIA1302 transgenic stains, the mRNA levels of *CrCIS* increased by 1003% to 1419%, whereas the enzyme activities of *CrCIS* increased by 209% to 266% (Figure 8). In summary, the overexpression of *CrCIS* gene encodes a key enzyme in the TCA cycle and causes photosynthetic carbon flux without fatty acid synthesis, thereby decreasing lipid synthesis in cells. The enhancement of CrCIS activity accelerated TCA and increased ATP synthesis, as demonstrated in the increased early growth rates of transgenic algae strains. Decreased lipid content was also observed using Nile red dye staining (Figure 9) and oil droplet counting (Additional file 1: Table S3). Fewer oil droplets were found in *CrCIS* overexpression transgenic algae than in the control.

### Expression of *CrCIS* in *E. coli* BL21 and detection of in vitro enzyme activities

**Figure 10:**
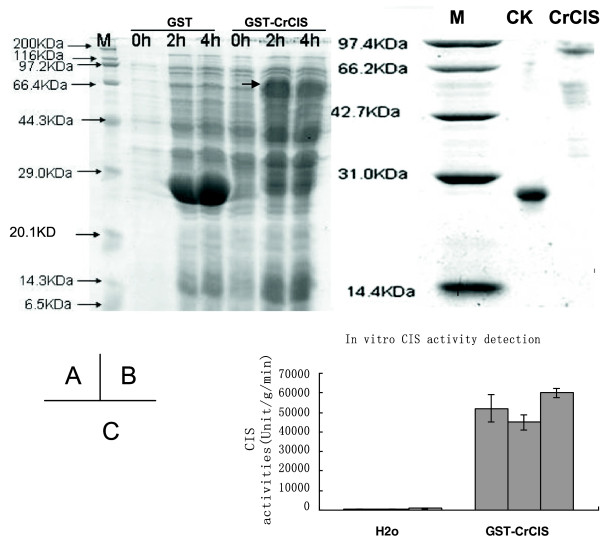
**Expressions of *CrCIS *gene in *E. coli *BL21 and enzyme activity detected in vitro.** After induced by IPTG and cultivated in 0, 2, 4, and 6 hours, total protein had been harvested and run through SDS-PAGE. **A)** Expression of *CrCIS* in *E. coli* BL21; **B)** Purified GST-CrCIS; **C)** Enzyme activity of GST-CrCIS detected in vitro.

Plasmids of pGEX-6p-1-CIS were constructed to express *CrCIS* in *E. coli* and verify enzyme activities. The recombinant vector was transformed into *E. coli* BL21 strain. Transformants were grown in LB medium and induced with 1 mM of isopropylthiogalactoside. The supernatant fraction of the denatured cells was loaded onto a 15% SDS-PAGE gel. A GST-CrCIS protein band of 75 kD was observed (Figure 10A). The fusion protein was purified with columns followed by enzyme activity assay (Figure 10B). Compared with the control, the enzyme activity of CIS increased 56-fold to 100-fold (Figure 10C). These behaviors indicated that the cloned gene of *CrCIS* exhibited biological activities.

## Discussion

Metabolic networks of photosynthetic eukaryotic organisms are complicated by the interplay of synthesis and degradation of carbohydrates, proteins, and lipids. Hence, the regulation of a main carbon metabolic pathway can affect other metabolic pathways. To increase lipid production, the synthesis of fatty acids of the substrates through a substitute method of metabolic pathway regulation should be improved. Studies on the relationships between carbon flux and lipid accumulation have focused on Arabidopsis thaliana and oil crops. Thus far, the genes involved were ACCase [11], sn-glycerol-3-phosphate dehydrogenase [12], pyruvate dehydrogenase kinase [13,14], pyuvate kinase complex [15,16], and d-glucose-6-phosphate dehydrogenase [17]. Ratcliffe and Shachar-Hill labeled carbon flux with isotope in rape seed development. They identified sugar cane and glucose as the main source of carbon flux during rape seed development [18]. This process produced TP, PAG, and PEP. The photosynthetic carbon flux was divided into four parts: 6.1% was used to synthesize OAA through PEP catalyzed by PEPC in the glycolytic pathway; 23.8% entered the TCA pathway through Pyr and Ac–CoA produced from PEP; 23.8% was used to synthesize amino acids through the intermediate products TP, PAG, and PEP; and the remaining 46.3% was used for lipid production through Ac–COA. Therefore, carbon efficiently flowed into lipid synthesis through directly or indirectly inhibiting PEPC activity, TCA pathway, and protein synthesis. Fan et al. identified starch as that the main storage sink for *Chlamydomonas* rather than oil. Even under N starvation conditions, the carbon flux derived from acetate has two destinations, namely, starch and oil. Significant increase in oil accumulation was caused by the blocking of starch biosynthesis in the starchless mutant BAFJ5. Thus, carbon supply was the limiting factor in TAG biosynthesis by *Chlamydomonas*[19]. In our study, the effective blocking of CrCIS activity through the RNA interference method increased lipid accumulation, whereas increased CrCIS activity in transgenic algal cells decreased lipid accumulation. CIS is one of the key enzymes involved in cell metabolism. This enzyme catalyzes acetyl–CoA and OAA to form citrate, the first reaction of TCA in mitochondria. This reaction uses most of the carbon flux. Modulating CIS activity is the most important step affecting carbon flux distribution in TCA. The activity of CIS determines the speed of TCA and the concentrations of PEP and pyruvic acid. Both PEP and pyruvic acid can enter the plastid and then transform into acetyl–CoA and propionyl–CoA, respectively. Acetyl–CoA and propionyl–CoA are substrates for fatty acid synthesis. In this study, *CrCIS* gene silencing increased TAG production by 169.5% in algal cells. CrCIS activity decreased by 16.7% to 37% in transgenic strains compared with that of the control. The mRNA levels of *DGAT2* and *PAP2* directly related to lipid biosynthesis significantly increased. This result proposed that carbon flux in lipid synthesis was altered in transgenic strains. Application of the same method in rape development is unsuitable, as determined by Tong et al. [4]. Antisense gene silencing in *CIS* was introduced to rape, decreasing the mRNA level of *CIS* but not its lipid production. The results obtained were contradictory with the expected low lipid production of seeds and leaves, as well as with the corresponding phenotypes such as dwarf, small lamina, and shortened pod. The association of this phenotype with the complexity of multicellular organisms was speculated because the *CIS* gene can function in rape development. The knockdown of *CIS* can affect the expression of genes related to cell differentiation and development. Nevertheless, our results showed that in unicellular photosynthetic eukaryote *Chlamydomonas*, *CrCIS* gene silencing did not slow down cell proliferation (Figure 3).

We observed that *CrCIS* gene silencing resulted in the artificial blockage of TCA pathways, which caused photosynthetic carbon flux to mainly enter the oil accumulation in *C. reinhardtii*. This observation is consistent with the hypothesis that carbon supply is the limiting factor in TAG synthesis by *Chlamydomonas*[19]. Further studies are required to elucidate the mechanisms underlying the exact signals triggering the switches. Our findings suggested that increasing oil by suppressing *CrCIS* expression in microalgae was feasible. Furthermore, the success of inducing oil accumulation by *CrCIS* silencing validated the usefulness and effectiveness of biotechnology methods in manipulating microalgae to promote oil production.

## Conclusions

In this study, *CrCIS* gene silencing increased TAG production by 169.5% in algal cells. CrCIS activity decreased by 16.7% to 37% in transgenic strains compared with that of the control. The mRNA levels of *DGAT2* and *PAP2* that are directly related to lipid biosynthesis significantly increased. Conversely, overexpression of *CrCIS* gene decreased the TAG level by 45% but increased CrCIS activity by 209% to 266% in transgenic algae. This result suggested that the silencing or overexpression of *CrCIS* gene caused photosynthetic carbon flux to enter fatty acid synthesis or the TCA cycle, thereby controling the lipid synthesis in cells.

## Methods

### Bioinformatics

Information on *Chlamydomonas* CIS gene (JGI Protein ID: 194915; NCBI XP_001695571) was obtained from the JGI *Chlamydomonas* database (*C. reinhardtii* v4.0 http://genome.jgi-psf.org/Chlre4/Chlre4.home.html). Subcellular localization of proteins was predicted by Euk-mPLoc 2.0 [20,21]. Sequence alignment and the phylogenetic tree of CIS was created using ClustalW (http://www.genome.jp/tools/clustalw/) [22]. Active consensus sites were identified based on the Sanger Pfam database (http://pfam.sanger.ac.uk/search).

### Algal strain, cultivation conditions, and biomass assay

*C. reinhardtii* CC425 (mt) was purchased from the *Chlamydomonas* Genetics Center at Duke University. Cells were grown on a Tris-acetate-phosphate (TAP) agar plate, inoculated into 100 mL Erlenmeyer flasks containing 50 mL of HSM and N-deficient HSM (HSM-N) media [23]. The HSM medium was composed of NH_4_Cl (0.500 g L^-1^), MgSO_4_ · 7H_2_O (0.020 g L^-1^), CaCl_2_ · 2H_2_O (0.010 g L^-1^), K_2_HPO_4_ (1.440 g L^-1^), KH_2_PO_4_ (0.720 g L^-1^), NaAc (2.000 g L^-1^), H_3_BO_3_ (0.001 g L^-1^), MnCl_2_ · 4H_2_O (0.005 g L^-1^), ZnSO_4_ · 7H_2_O (0.022 g L^-1^), FeSO_4_ · 7H_2_O (0.005 g L^-1^), CoCl_2_ · 6H_2_O (0.002 g L^-1^), Na_2_Mo_7_O_24_ · 4H_2_O (0.002 g L^-1^), and Na_2_ · EDTA (0.050 g L^-1^). The HSM-N medium contained the same components but with NH_4_Cl instead of NaCl. All cultures were maintained in an incubator shaker (230 rpm at 25°C) and then exposed to continuous illumination at a light intensity of 150 μmol · m^-2^ · s^-1^.

Biomass concentration (g/L) was determined by measuring the optical density of the samples at 490 nm (OD_490_) as described in a previous study [24]. To generate the standard curve, a series of *C. reinhardtii* CC425 samples with different biomass concentrations were collected. The OD_490_ and cell dry weight were gravimetrically determined using dried cells to plot the standard curve of OD_490_ versus biomass concentration (g/L). Samples were diluted to appropriate ratios to ensure that the measured OD490 values ranged from 0.15 to 0.75, if applicable. Biomass concentration was then calculated using the following formula: cell dry weight (g/L) = 0.7444 × OD_490_ – 0.0132 (Additional file 1: Figure S1).

### Lipid content analysis

The Nile Red fluorescence method and GC/MS were used to determine lipid and TAG levels [24,25]. Algal cells were directly stained with 0.1 mg/mL Nile Red for 10 min, and then fluorescence was measured at excitation and emission wavelengths of 470 and 570 nm, respectively. The fluorescence value was calculated using the Equation FD (470/570) = (*A*_2_ – *A*_1_), where A_2_ is the fluorescence value of algal cells after staining with Nile Red, and *A*_1_ is that of algal cells before staining (Additional file 1: Figure S2). Total lipid extraction was carried out according to a modified method. Logarithmic-phase algal cells were collected by centrifugation and extracted using an extraction buffer (methanol : chloroform : methanoic acid, 2 : 1 : 0.1), 1 M KCl, and 0.2 M H_3_PO_4_. The lipids were obtained by centrifugation at 13780 *g* for 3 min. For TAG separation, we used Si60 silica TLC plates for thin-layer chromatography. The TLC plates were immersed in 0.15 M (NH4)_2_SO_4_ for 30 s and stored in an airtight container for two days. These plates were then placed in an oven at 120°C for 2.5 h and cooled at room temperature. Samples were then placed under N_2_ flow, and TAGs were observed on TLC plates through iodine staining. Lipid analysis was conducted as previously described. Fatty acid methyl esters derived from TAG were analyzed through GC/MS [26]. For the microscopic assay, images were acquired using a Nikon 80i Fluorescence Microscope after cells were stained with Nile Red. Nile Red signals were captured using an excitation wavelength of 480 nm, and emission was collected between 560 nm and 600 nm [27-29]. Thirty cell lipid droplets from each algal strain were examined to determine the difference between lipid contents.

### RNA extraction

Total RNA was prepared as described by Li et al. with some modifications [30]. Cells from 10 mL of cultivated algae were collected by centrifugation at 10,000 × g for 1 min. After a series of phenol–chloroform extractions, nucleic acids were precipitated with two volumes of absolute ethanol and then washed with 75% ethanol. Finally, the air-dried pellet was dissolved in 40 μL of RNase-free water. RNA concentration was determined through spectrophotometry, and the integrity was examined through agarose gel electrophoresis.

### Cloning of *CrCIS* gene

First-strand cDNA was synthesized using SuperScriptTM III Reverse Transcriptase (Invitrogen, USA) according to the instructions of the manufacturer. A fragment of the *CrCIS* gene was amplified by PCR using the primers CrCISL: 5′-TACTTGGCCCGTGCCTGTAT-3′ and CrCISR: 5′-CCTCCCTCCTTCATGTGTGT-3′. PCR reactions were performed in a final volume of 25 μL containing 1× PCR reaction buffer, 2 mM MgCl2, 0.4 μmol of each primer, 0.25 mM dNTPs, 1 μL DMSO, 0.5 M Betain, and 0.5 U Taq DNA polymerase (Promega, USA) according to the following program: 4 min at 95°C; 35 cycles of denaturation for 40 s at 95°C, annealing for 40 s at 58°C, and elongation for 20 s at 72°C; and 10 min at 72°C. After purification using the EZ-10 Spin Column DNA Gel Extraction Kit (BBI, Canada), DNA was inserted into vector pMD18-T following the instructions of the manufacturer (TaKaRa, Japan). The resulting plasmid was designated as pMD18T-CrCIS. The sequences of the cloned *CrCIS* gene were verified through double-stranded sequence analysis (Shanghai Sangon Biological Engineering Technology & Services Co., Ltd).

### Construction of the RNAi vector against *CrCIS* gene

To construct the RNAi vector against *CrCIS* gene, a fragment of *C. reinhardtii* 18S gene was amplified with primers 5′-CGAACTTCTGCGAAAGCAT-3′ and 5′-TCAGCCTTGCGACCATACT-3′ and then inserted into pMD18-T producing pMD18T-18S. The fragment of CrCIS and its reverse complementary sequences were amplified through PCR using pMD18T-CrCIS as the template and the primers CISRNAiL: 5′-GACGCGCACAGCGGCGTGCT-3′ and CISRNAiR: 5′-CCTCCCTCCTTCATGTGTGT-3′. The PCR fragment was then digested with KpnI/BamHI and HindIII/SalI, after which the fragment was inserted into the corresponding cloning sites of pMD18T-18S. The fragment yielded pMD18-CrCIS F-18S-CrCIS R, which contained an inverted repeat sequence of CrCIS (CrCIS IR). pMD18-CrCIS F-18S-CrCIS R was double digested with KpnI and HindIII to obtain *CrCIS* IR. Finally, *CrCIS* IR was inserted as a blunt-end fragment into EcoRI-digested pMaa7/XIR to produce pMaa7IR/CrCIS IR.

### Construction of the overexpression vector of *CrCIS* gene for *Chlamydomonas*

To construct the overexpression vector of *CrCIS* gene, the coding sequence of *CrCIS* was amplified through PCR using pMD18T-CrCIS and primers 5′-TTCAAGATCTGATGCTGGCCACGGCC-3′ and 5′-TAACACTAGTTTACGCGGACTGGCC-3′ as templates. The fragment was digested with NcoI/SpeI and inserted into similarly digested pCAMBIA1302 to yield pCAMBIACIS facilitating the overexpression of *CrCIS*.

### Transformation of *Chlamydomonas*

Transformation of *C. reinhardtii* strain CC425 was performed as described by Kindle [31]. *C. reinhardtii* cells were grown in TAP medium until having a cell density of 1–2 × 10^6^ cells/mL. Cells were collected by centrifugation, washed twice, and then resuspended in TAP medium until having a cell density of approximately 1 × 10^8^ cells/mL. Plasmid DNA was introduced into the cells through the glass bead procedure. In each case, 2 μg of plasmid DNA was added to a mixture containing 400 μl of cells, 100 μl of 20% polyethylene glycol, and 300 mg of sterile glass beads. The reaction was mixed for 15 s on a benchtop vortex. To enable induction of RNAi or gene expression, cells were allowed to recover for one day before plating onto selective media. RNAi transformants were selected on TAP medium containing 1.5 mM l-tryptophan, 5 μg/mL paromomycin, and 5 μM 5-FI. Meanwhile, pCAMBIACIS transformants were selected on TAP medium containing 50 μg/mL hygromycin. Plates were incubated under dim light (approximately 50 μmol · m^-2^ · s^-1^ photosynthetically active radiation), and isolated transgenic strains were kept under constant selective pressure.

### Expression of *CrCIS* in *Escherichia coli* BL21

To express *CrCIS* in *E. coli* BL21, the coding region was amplified from pMD18T-CrCIS with primer pairs GEXCIS-F: 5′-TAAAGGATCCATGCTGGCCACGGCC-3′ and GEXCIS-R: 5′-TAAGCTCGAGTTACGCGGACTGGCC-3′. The amplified fragments were digested with BamHI/SalI and then inserted into similar digested pGEX-6p-1 to administer pGEXCIS. The transformation of *E. coli* BL21 and subsequent foreign protein detection by sodium dodecyl sulfate polyacrylamide gel electrophoresis (SDS-PAGE) and purification of the glutathione S-transferase (GST)-fusion protein were conducted as described by Sambrook and Russell [32].

### Quantitative real-time PCR

Samples for real-time PCR analysis were performed as described by Fei et al. and Deng et al. [10,33]. RNA was extracted using TRIzol Reagent (Shanghai Sangon Biological Engineering Technology & Service Co.). Single-strand cDNA was synthesized using an Invitrogen SuperScriptTM III cDNA synthesis kit with 100 ng of RNA, and random primers performed at 65°C for 5 min, 25°C for 5 min, and 42°C for 50 min. Real-time PCR was performed on a BioRad iCycler iQ Real-Time PCR Detection System using SYBR Green as the fluorescent dye. Each reaction was performed with a final volume of 25 μl with the following components: 0.2 pmol of each primer, 1 μl of cDNA, and 12.5 μl of SYBR Green Mix (Invitrogen SYBR GreenER qPCR). Water was used to adjust the volume to 25 μl. The iCycler protocol was performed as follows: denaturing at 95°C, 5 min, and 40 cycles of denaturing at 95°C for 30 s; annealing at 54°C for 30 s; and amplification at 72°C for 15 s. The specificity of PCR amplification was examined with a melting curve program (55°C to 100°C at a heating rate of 0.5°C/s). 18S rRNA was used as the control with primers 18SrRNAF (5′-TCAACTTTCGATGGTAGGATAGTG-3′) and 18SrRNAR (5′-CCGTGTCAGGATTGGGTAATTT-3′). The expression of this control gene was measured and concluded to be constant under all conditions used in this study. The gene-specific primers listed in Table S2 (Additional file 1) were used to evaluate the quantity of target cDNA. The amplification rate of each transcript (Ct) was calculated through the PCR baseline-subtracted method performed with iCycler software at a constant fluorescence level. Ct values were determined over three repeats. Relative fold differences were calculated based on the relative quantification analytical method (2-ΔΔCT) using 18 s rRNA amplification as an internal standard [34].

### Detection of CIS activities

Transgenic algal samples cultivated in the log phase were collected and centrifuged (3000 rpm for 5 min). Algal cell pellets were then collected and washed with phosphate buffer. Cells were sonicated in 3 mL of extract buffer and then centrifuged (3000 rpm for 5 min) to collect the supernatant totaling a volume of 6 mL through adding extract buffer. The enzyme extract to be detected was obtained by dialyzing with extract buffer. CIS activity was measured as described by Sienkiewicz et al. with some modifications [35]. The dialyzed enzyme sample was mixed with 40 mmol/L Tris–HCl buffer (pH 9.0), 0.04 mmol/L DTNB, 0.08 mmol/L acetyl–CoA, and 0.04 mmol/L OAA. The absorbance of the samples requiring 15 s per scan was measured, and the total scan time was 3 min.

## Competing interests

The authors declare that they have no competing interests.

## Authors’ contributions

XDD executed the molecular biology studies, participated in the data analysis and drafted the manuscript. JJC performed the lipid and TAG content detection, participated in the SDS-PAGE and CIS activity detection. XWF accomplished the statistical analysis, conceived the study, participated designing the study, coordinated the research, and assisted in drafting the manuscript. All authors read and approved the final manuscript.

## Supplementary Material

Additional file 1: Figure S1 The correlation between biomass (Cell dry weight g/L) and the optical density OD490. **Figure S2:** The correlation between lipid concentration (Triolein mg/20 mL) and the fluorescence value FD470/570. **Table S1:** Primers used in this work. **Table S2:** Primers for Real Time PCR. **Table S3:** The numbers of lipid droplets in algae cell. Click here for file
