# Supplementary material for: Effect of the expression and knockdown of citrate synthase gene on carbon flux during triacylglycerol biosynthesis by green algae Chlamydomonas reinhardtii
Source: BMC Biochem. 2013 Dec 30;14:38. doi: 10.1186/1471-2091-14-38 (PMC3890626; doi:10.1186/1471-2091-14-38)
Supplement: Additional file 1: Figure S1 — The correlation between biomass (Cell dry weight g/L) and the optical density OD490. Figure S2: The correlation between lipid concentration (Triolein mg/20 mL) and the fluorescence value FD470/570. Table S1: Primers used in this work. Table S2: Primers for Real Time PCR. Table S3: The numbers of lipid droplets in algae cell. [file 1471-2091-14-38-S1.doc]

Supplementary data Figure 1 the correlation between biomass (Cell dry weight g/L) and the optical density OD490

Supplementary data Figure 2 the correlation between lipid concentration (Triolein mg/20mL) and the fluorescence value FD470/570

Supplementary data Table 1 primers used in this work

| Primers for RNAi construction | |
| --- | --- |
| Gene | Primer sequence |
| *CrCIS* | 5’-GACGCGCACAGCGGCGTGCT -3’  5’- CCTCCCTCCTTCATGTGTGT-3’ |
| Primers for full length gene clone | |
| Gene name | Primer sequence |
| *CrCIS* | 5’-TACTTGGCCCGTGCCTGTAT-3’  5’-CCTCCCTCCTTCATGTGTGT-3’ |
| Primers for overexpression in Chlamydomonas | |
| Gene name | Primer sequence |
| *CrCIS* | 5’-TTCAAGATCTGATGCTGGCCACGGCC-3’  5’-TAACACTAGTTTACGCGGACTGGCC-3’ |
| Primers for expression in E.coli | |
| Gene name | Primer sequence |
| *CrCIS* | 5’-TAAAGGATCCATGCTGGCCACGGCC-3’  5’- TAAGCTCGAGTTACGCGGACTGGCC -3’ |

Supplementary data Table2 Primers for Real Time PCR

| Gene | Primer sequence |
| --- | --- |
| *CrCIS* | 5’-GACGCGCACAGCGGCGTGCT -3’  5’- CCTCCCTCCTTCATGTGTGT-3’ |
| *CrDGAT2* | 5’-GCTGGCTTCAAACTTCTGG-3’  5’-CACCTTGTCCACCTGTATGG-3 |
| *CrPAP2* | 5’-GCGTGTTTGCCTACTTCCTC-3’  5’-CACTACTCGCGCCGTACAT-3 |

Supplementary data Table 3 The numbers of lipid droplets in algae cell.

| Algae Strains | Numbers of the lipid droplets/Cell |
| --- | --- |
| Maa7-4 | 7±3 |
| Maa7-10 | 6±2 |
| Maa7-19 | 7±2 |
| CIS-RNAi-1 | 12±5 |
| CIS-RNAi-28 | 16±4 |
| CIS-RNAi-65 | 15±2 |
| pCAMBIA2 | 10±3 |
| pCAMBIA8 | 9±2 |
| pCAMBIA16 | 12±3 |
| pCAMCIS13 | 5±1 |
| pCAMCIS28 | 4±1 |
| pCAMCIS54 | 7±2 |

Maa7-4(10,19), pMaa7IR/XIR transgenic algae strains; CIS-RNAi-1(28,65), pMaa7IR/CrCISIR transgenic algae strains; pCAMBIA-2(8,16), pMCAMBIA1302 transgenic algae strains; pCAMCIS-13(28,54), pCAMCIS transgenic algae strains.
